# No evidence that polymorphisms of brain regulator genes *Microcephalin* and *ASPM* are associated with general mental ability, head circumference or altruism

**DOI:** 10.1098/rsbl.2006.0586

**Published:** 2007-01-23

**Authors:** J. Philippe Rushton, Philip A Vernon, Trudy Ann Bons

**Affiliations:** The University of Western OntarioOntario, Canada N6A 3C2

**Keywords:** altruism, brain size, genes, general intelligence, social intelligence

## Abstract

We test the hypothesis that polymorphisms of the brain regulator genes *MCPH1* and *ASPM* contribute to variations in human brain size and its correlates. We measured general mental ability, head circumference and social intelligence in 644 Canadian adults (496 Caucasians, 36 Orientals, 84 Mixed Race/Other and 28 Blacks; 257 men and 387 women). The gene polymorphisms were assessed from buccal DNA; mental ability by Wonderlic Personnel Test and Multidimensional Aptitude Battery; head circumference by stretchless tape; and social intelligence by prosocial attitude questionnaires. Although all measures were construct valid and the allele frequencies showed expected population differences, no relationship was found between the genes and any of the criteria. Among Caucasian 18–25 year olds, for example, the two mental ability tests correlated with each other (*r*=0.78, *N*=476, *p*<0.001), with head circumference (*r*=0.17, *N*=182, *p*<0.05) and with prosocial attitudes (*r*=0.23, *N*=182, *p*<0.001).

## 1. Introduction

Two newly discovered genes, *Microcephalin* (*MCPH1*) on chromosome 8p23 and abnormal spindle-like microcephaly associated (*ASPM*) on chromosome 1q31, attracted much attention when reported to be (i) associated with autosomal recessive primary microcephaly, (ii) positively accelerated in molecular evolutionary rate through the simian line leading to *Homo sapiens*, and (iii) under recent positive selection in modern humans (Evans *et al*. 2005; Mekel-Bobrov *et al*. 2005). The *MCPH1* allele favoured by selection in modern humans, known as the D (derived) allele, was estimated to have arisen approximately 37 000 years ago (95% CI: 60 000–14 000 years ago), about the time symbolic behaviour became widespread in Europe, while the favoured D allele for *ASPM* was estimated to have arisen approximately 5800 years ago (95% CI: 14 000–500 years ago), about the time cities developed in the Near East. The D alleles for both *MCPH1* and *ASPM* now exist in high frequency. Both genes were hypothesized to confer a selective advantage such as increased brain size and general mental ability (GMA).

GMA correlates approximately *r*=0.20 with external head circumference and *r*=0.40 with MRI measured brain volume (Vernon *et al*. 2000; Rushton & Ankney 2007). Moreover, heritabilities of 50–80% are found for both brain size and GMA. For more than 10 000 pairs of identical and same sex fraternal twins living together, the mean correlation for GMA is 0.86 and 0.60, respectively; for more than 27 000 pairs of siblings, it is 0.49; and for identical twins raised apart it is almost as high as for identical twins reared together, *r*=0.78 for 93 pairs (Bouchard & McGue 2003). One MRI study of 112 extended twin families found heritabilities of 82% for whole-brain grey matter volume, 87% for whole-brain white matter volume and 86% for general intelligence (Posthuma *et al*. 2002). It reported that the correlation between grey-matter volume and GMA, as with white-matter volume and GMA, was completely due to genetic factors and not due to environmental factors. The purpose of the present study was to investigate the hypothesis of an association between the two microcephaly alleles and GMA, head size and social intelligence.

## 2. Material and methods

Participants were 644 Canadian adults (496 Caucasians, 36 Orientals, 84 Mixed Race/Other and 28 Blacks; 257 men and 387 women). They were paid Canadian $40 (approx. US $32) for a 2-h testing session. Between February and April 2005, 405 people were recruited from a university subject pool and by local advertisements; another 239 were later recruited from an employment agency.

GMA was assessed by the 50-item Wonderlic Personnel Test (Wonderlic Personnel Test 1992) and the 10 subtests (five verbal and five performance) of the Multidimensional Aptitude Battery (MAB; Jackson 1984). Head circumference correlates about 0.60 with MRI brain volume (Rushton & Ankney 2007) and was measured using a stretchless tape along the maximal plane of the skull just above the ear. Social intelligence, a promissory but problematic concept (Visser *et al*. 2006), was assessed by two prosocial attitude scales, also found to be heritable (Rushton 2004): the 20-item Self-Report Altruism Scale (Rushton *et al*. 1981) and the 20-item Arizona Mini-*K* Scale (Figueredo *et al*. 2004). Head circumference and Mini-*K* were assessed only in the second sample.

One of the authors (Trudy Ann Bons) administered the tests in small groups. Participants completed questions on handedness, demographic background and social intelligence, then the Wonderlic (12 min) and the 10 MAB subtests (7 min each). Finally, they provided two DNA buccal swabs, one from inside each cheek. The DNA was genotyped according to standard procedures as published previously. Three genotypes of the *MCPH1 G37995C* and *ASPM A44871G* SNPs (where C and G are diagnostic of the D allele, respectively) were coded: AA for the homozygous non-D allele, AD for the heterozygous alleles and DD for the homozygous D allele.

## 3. Results

Null results were found across all ways of examining the 644 participants, despite evidence showing the measures were construct valid. The allele frequencies, means and s.d.s of the measures are shown in table 1. The D allele of *MCPH1* had an overall frequency of 84% and was in Hardy–Weinberg equilibrium among all population groups and among Caucasians was more often in homozygote than heterozygote form. Similarly, the D allele of *ASPM* had an overall frequency of 32% and was in Hardy–Weinberg equilibrium among all population groups and among Caucasians was more often in heterozygote and less often in homozygote form. These are similar to the initial reports by Evans *et al*. (2005) and Mekel-Bobrov *et al*. (2005).

The psychological tests also showed properties similar to previous research. For the Caucasian sample between the ages of 18 and 25, the two tests of GMA correlated with each other (*r*=0.78, *N*=476, *p*<0.001) and, as an aggregate *g* factor, with head circumference (*r*=0.17, *N*=182, *p*<0.05), Altruism (*r*=0.17, *N*=476, *p*<0.001) and Mini-*K* (*r*=0.23, *N*=182, *p*<0.001). The two prosocial attitudes scales correlated with each other (*r*=0.25, *N*=182, *p*<0.001), but not with head circumference. (Similar results were found for the entire sample; e.g. between GMA and head circumference, *r*=0.19, *N*=239, *p*<0.001.) Regardless of sample, a principal components analysis of the Wonderlic and the 10 MAB subtests showed loadings of 0.69 or higher on the first factor. Each individual's *g* factor score was also calculated based on the first component as this factor is the one most likely to show a genetic effect (Plomin & Kovas 2005).

To eliminate the confounding effect of population stratification, our main analysis was limited to Caucasians, which are by far the largest of our constituent samples. Figures 1 and 2 show the distribution of the AA, AD and DD alleles for the two genes with various criteria. No predicted relation was found between either of the two genes and any of the dependent variables (*F*_S_<1.00). Neither eliminating the heterozygotic AD category nor combining it with the DD category showed the AA versus DD comparisons to be significant (*F*_S_<1.00). Nor did the effect emerge when examining the relation in a variety of other ways including combining the two genes, or all the samples.

## 4. Discussion

No evidence was found of a relation between the two candidate genes *ASPM* and *MCPH1* and individual differences in head circumference, GMA or social intelligence. The absence of evidence was noted even in the analysis of the full sample (*N*=644) despite stratification effects due to ethnicity that would have biased the results in favour of finding a genetic effect (Blacks had more ancestral AA genes and lower IQ scores). Null findings can arise due to procedural problems, but in this study, the variables showed construct validity and expected measurement properties. Moreover, the null results join one other based on 120 MRI-measured brain volumes (Woods *et al*. 2006). Uncertainty surrounding the microcephalin hypothesis is thereby reduced.

Thousands of genes are expressed in the brain and null findings are common. Moreover, a sample of 496 (or even the 644 available for the entire sample) provides only 95% power to exclude an association that accounts for 2% of the variance. Given that genetic effect sizes turn out to be extremely small, typically 0.1%, and contribute interchangeably and additively (Plomin *et al*. 2006), most studies have been seriously underpowered to detect and replicate effects. Association studies of many markers in thousands of individuals may be required to identify appropriate genes.

## Figures and Tables

**Figure 1:**
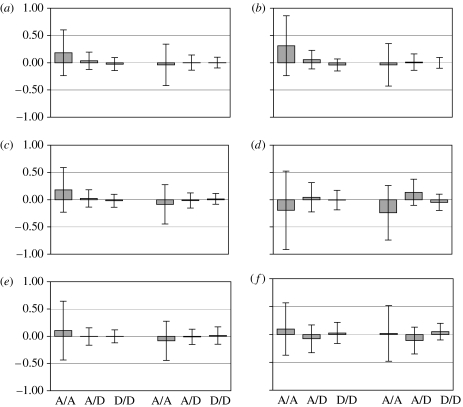
Relation (*z*-score and 95% CI) of *MCPH1* gene variants (AA, AD and DD) to (*a*) Multidimensional Aptitude Battery for Caucasians (*N*_S_=15, 137, 300) and total sample (*N*_S_=31, 186, 367); (*b*) Wonderlic Personnel Test for Caucasians (*N*_S_=15, 137, 300) and total sample (*N*_S_=31, 186, 367); (*c*) *g* factor scores for Caucasians (*N*s =15, 137, 300) and total sample (*N*_S_=31, 186, 367); (*d*) head circumference for Caucasians (*N*_S_=7, 50, 121) and total sample (*N*_S_=10, 72, 151); (*e*) Self-Report Altruism Scale for Caucasians (*N*_S_=15, 137, 300) and total sample (*N*_S_=31, 186, 367); and (*f*) Mini-*K* scale for Caucasians (*N*_S_=7, 50, 121) and total sample (*N*_S_=10, 72, 151).

**Figure 2:**
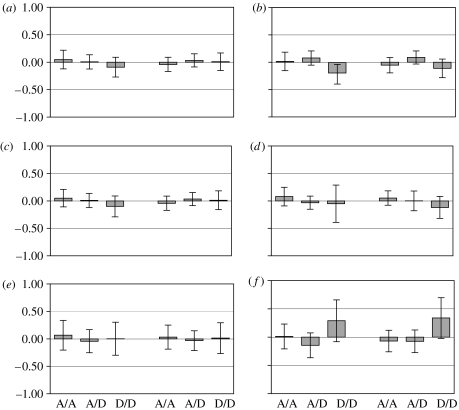
Relation (*z*-score and 95% CI) of *ASPM* gene variants (AA, AD and DD) to (*a*) Multidimensional Aptitude Battery for Caucasians (*N*_S_=150, 231, 92) and total sample (*N*_S_=237, 275, 102); (*b*) Wonderlic Personnel Test for Caucasians (*N*_S_=150, 231, 92) and total sample (*N*_S_=237, 275, 102); (*c*) *g* factor scores for Caucasians (*N*_S_=150, 231, 92) and total sample (*N*_S_=237, 275, 102); (*d*) head circumference for Caucasians (*N*_S_=59, 81, 38) and total sample (*N*_S_=93, 99, 41); (*e*) Self-Report Altruism Scale for Caucasians (*N*=150, 231, 92) and total sample (*N*_S_=237, 275, 102); and (*f*) Mini-*K* scale for Caucasians (*N*_S_=59, 81, 38) and total sample (*N*_S_=93, 99, 41).

**Table 1:** Sample size, mean (s.d.) and frequency of variables by population group and sex.

	Caucasian		Oriental		Mixed/Other		Black	
				
	men	women	men	women	men	women	men	women
*variable* (*N*)	*193*	*303*	*13*	*23*	*38*	*46*	*13*	*15*
Wonderlic IQ	105 (12)	105 (11)	100 (13)	104 (13)	99 (13)	104 (12)	102 (17)	98 (10)
MAB IQ	105 (14)	104 (12)	102 (17)	103 (12)	99 (14)	100 (13)	96 (20)	94 (9)
*g* factor score	0.1 (1.0)	0.1 (0.9)	−0.2 (1.2)	0.1 (0.9)	−0.4 (1.1)	−0.2 (1.0)	−0.5 (1.4)	−0.7 (0.8)
altruism	56 (10)	55 (9)	55 (8)	54 (8)	52 (10)	55 (11)	52 (9)	54 (10)
*variable* (*N*)	*82*	*102*	*5*	*5*	*16*	*18*	*5*	*6*
Mini-*K*	17 (12)	29 (11)	21 (8)	10 (16)	18 (16)	24 (12)	23 (14)	31 (9)
head (cm)	58 (2)	56 (2)	58 (2)	57 (1)	58 (2)	56 (2)	57 (2)	59 (3)
*variable* (*N*)	*179*	*273*	*12*	*18*	*34*	*43*	*11*	*14*
*MCPH1* A/A%	3	3	8	5	15	2	18	43
*MCPH1* A/D%	28	32	42	28	47	33	55	21
*MCPH1* D/D%	69	65	50	67	38	65	27	36
*variable* (*N*)	*186*	*287*	*12*	*22*	*37*	*44*	*12*	*14*
*ASPM* A/A%	31	32	50	86	43	59	58	93
*ASPM* A/D%	51	47	50	14	51	23	42	7
*ASPM* D/D%	18	21	0	0	6	18	0	0
